# Simultaneous Labyrinthectomy and Cochlear Implantation for Patients with Single-Sided Ménière's Disease and Profound Sensorineural Hearing Loss

**DOI:** 10.1155/2015/457318

**Published:** 2015-08-24

**Authors:** G. Doobe, A. Ernst, R. Ramalingam, P. Mittmann, I. Todt

**Affiliations:** ^1^Department of Otolaryngology, Head and Neck Surgery, Unfallkrankenhaus Berlin, 12683 Berlin, Germany; ^2^KKR ENT Hospital & Research Institute, Chennai 600010, India

## Abstract

*Objective.* To investigate the treatment outcome of a simultaneous labyrinthectomy and cochlear implantation in patients with single-sided Ménière's disease and profound sensorineural hearing loss. *Study Design.* Prospective study. *Method.* Five patients with single-sided Ménière's disease with active vertigo and functional deafness were included. In all cases, simultaneous cochlear implantation combined with labyrinthectomy surgery was performed. The outcome has been evaluated by the Dizziness Handicap Inventory (DHI) and speech recognition. *Results.* The combined labyrinthectomy and cochlear implantation led in all patients to a highly significant reduction of dizziness up to a *restitutio ad integrum*. After activation of the cochlear implant and rehabilitation, a mean monosyllabic speech understanding of 69% at 65 dB was observed. *Conclusion.* For patients with single-sided Ménière's disease and profound sensorineural hearing loss the simultaneous labyrinthectomy and cochlear implantation are efficient method for the treatment of vertigo as well as the rehabilitation of the auditory system.

## 1. Introduction

Most cases of Ménière's disease can be successfully treated through conservative approaches to reach a sufficient suppression of vertigo symptoms and to maintain a satisfying quality of life. But patients without adequate control of vertigo and with severe to profound hearing loss present a therapeutical problem. In these cases, a variety of other invasive approaches of vertigo control exist, such as intratympanic steroids, intratympanic gentamicin therapy, endolymphatic sac decompression/saccotomy, labyrinthectomy, and vestibular nerve section. But with increasing effectiveness of vertigo control the treatment also leads to increased loss of function of the inner ear [1].

If the disease itself has already caused profound sensorineural hearing loss, there is no contraindication for a radical operative labyrinth-intervention. So it is obvious to combine invasive vertigo treatment with implantable hearing systems in patients after previously unsuccessful treatment. Labyrinthectomy has manifested itself as an efficient treatment in controlling attacks of vertigo [1]. For this reason, this study examines the success of simultaneous labyrinthectomy for the therapy of vertigo and cochlear implantation for the rehabilitation of the auditory system. This treatment has been described for the first time in a single patient by Zwolan and coworkers [2]. MacKeith et al. [1] described patients with bilateral Ménière's disease. For our opinion for the elimination of the ipsilateral vestibular function by labyrinthectomy, a proper function of the vestibular function of the contralateral side must be provided to exclude the risk of a Dandy syndrome. For this purpose extensive preoperative diagnostic investigation and a long-term evaluation are necessary to minimize the risk of a bilateral vestibulopathy.

## 2. Materials and Methods

Three female and two male patients were treated for single-sided Ménière's disease based on the American Association of Head and Neck Surgery criteria. The mean age was 61 years with a range from 46 years to 76 years. Duration of vertigo attacks was between 2 years and 25 years. As the most restrictive symptom the patients reported heavy attacks of vertigo several times a week and up to 20 times a day in the worst case.

All patients were treated using the same surgical technique of simultaneous labyrinthectomy and cochlear implantation between March and September of 2014. Surgery included the removal of the semicircular canals and removal of the otolith organs. The vestibule was filled with a muscle plug followed by bone pate coverage and fibrin glue. Cochlear implantation was performed by a posterior tympanotomy and a round window approach to the cochlea.

For a subjective evaluation, the Dizziness Handicap Inventory (DHI) questionnaire was used for the assessment of the vertigo control preoperatively as well as directly postoperatively, 6–8 weeks and 6 months after surgery. The DHI is a validated and reliable instrument for the estimation of dizziness and is commonly used for the functional outcome-evaluation [3]. The DHI was evolved for the self-perceived degree of impairment as a result of the postoperative symptom dizziness [4].

In addition the audiologic results of the cochlear implantation were determined by speech recognition by Freiburg numbers and monosyllabic words. Before the treatment of labyrinthectomy, the unilateral inner ear affection of the operated ear was proved by synopsis of all diagnostic instruments (pure tone audiogram, caloric function, cVEMP, and EcochG) in order to minimize the risk of a bilateral vestibulopathy. The study was approved by the IRB (*IRB- UKB-HNO-2014/1*).

## 3. Results

The DHI indicated distinctive functional, physical, and emotional problems in all cases. The mean preoperative deficit was assessed to be serious concerning the physical (DHI 22.4) and emotional (DHI 19.2) impairment as well as moderate concerning the functional impairment (DHI 11.6) (Table 1).

**Figure 1:**
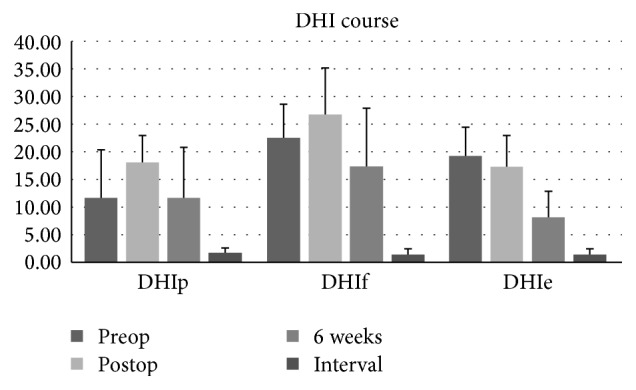
Time course of mean DHI score.

As shown in Figure 1, after surgical intervention the mean DHI followed a certain time course. Immediately postoperatively every patient reported an enhancement of symptoms. Initially, most patients were not capable of independent standing or movement. Walking was only possible with help. Symptoms of vertigo combined with nausea appeared with the slightest head movement. At this point the emotional burden showed no significant personal relief.

After 6 weeks the condition of the patients regarding the functional deficit and emotional impairment due to vertigo symptoms had already improved compared to the preoperative status. All patients reported only slight permanent dizziness and insecure walking. In all cases periods of vertigo attacks stopped from the day of the operation.

After an interval of 6 months all patients were almost symptom-free. Signs of vertigo only appeared with challenging activities, such as sport or hard physical work. Ordinary everyday activities were possible without any restrictions.

Before treatment all patients suffered from profound sensorineural hearing loss in the audiological evaluation. In the preoperative pure tone audiometry test a bone conduction could not be achieved at all or was only achieved with sound pressure levels higher than 70 dB. After cochlear implantation and the stage of auditory rehabilitation all patients reported a satisfying subjective ability of hearing. The individual speech recognition is shown in Table 2.

A one-way repeated measures ANOVA was conducted to determine whether there were statistically significant differences in DHI scores over the course of a 6-month postoperative follow-up period. For the DHIp scores, there were no outliers and the data was normally distributed, as assessed by boxplot and Shapiro-Wilk test (*P* > .05), respectively. The assumption of sphericity was not violated, as assessed by Mauchly's test of sphericity, *χ* ^2^(2) = 8.424, *P* = .157. Labyrinthectomy and cochlear implantation elicited statistically significant changes in DHIp scores, *F*(3,12) = 7.978, *P* = .003, and partial *η* ^2^ = .666, with DHIp scores increasing from 11.60 ± 8.65 preoperatively to 18.00 ± 4.90 postoperatively and decreasing to 11.60 ± 9.09 at 6 weeks and 2.80 ± 2.28 at 6 months after labyrinthectomy and cochlear implantation. Post hoc analysis with a Bonferroni adjustment revealed that DHIp scores decreased statistically significantly from postoperative to 6 months (15.2 (95% CI, 4.75 to 25.65) *P* = .013) (Table 3).

Similarly, for the DHIf score a one-way repeated measures ANOVA was conducted to determine whether there were statistically significant differences in DHIf scores over the course of a 6-month postoperative follow-up period. There were no outliers and the data was normally distributed, as assessed by box plot and Shapiro-Wilk test (*P* > .05), respectively. The assumption of sphericity was not violated, as assessed by Mauchly's test of sphericity, *χ* ^2^(2) = 7.663, *P* = .200. Labyrinthectomy and cochlear implantation elicited statistically significant changes in DHIf scores, *F*(3,12) = 18.149, *P* < .001, and partial *η* ^2^ = .819, with DHIf scores increasing from 22.40 ± 6.23 preoperatively to 26.80 ± 8.32 postoperatively and decreasing to 17.20 ± 10.73 at 6 weeks and 4.40 ± 4.34 at 6 months after labyrinthectomy and cochlear implantation. Post hoc analysis with a Bonferroni adjustment revealed that DHIf scores decreased statistically significantly from preoperative to 6 months (18.00 (95% CI, 8.30 to 27.70) *P* = .005) and postoperative to 6 months (22.40 (95% CI, 12.51 to 32.83) *P* = .002).

For the DHIe score a one-way repeated measures ANOVA was conducted to determine whether there were statistically significant differences in DHIe scores over the course of a 6-month postoperative follow-up period as well. There were no outliers and the data was normally distributed, as assessed by box plot and Shapiro-Wilk test (*P* > .05), respectively. The assumption of sphericity was not violated, as assessed by Mauchly's test of sphericity, *χ* ^2^(2) = 4.407, *P* = .517. Labyrinthectomy and cochlear implantation elicited statistically significant changes in DHIe scores, *F*(3,12) = 21.283, *P* < .001, and partial *η* ^2^ = .842, with DHIe scores decreasing from 19.20 ± 5.22 preoperatively to 17.20 ± 5.76 postoperatively to 8.00 ± 4.69 at 6 weeks and  .80 ± 1.10 at 6 months after labyrinthectomy and cochlear implantation. Post hoc analysis with a Bonferroni adjustment revealed that DHIe scores decreased statistically significantly from preoperative to 6 months (18.40 (95% CI, 8.99 to 27.81) *P* = .004) and postoperative to 6 months (16.40 (95% CI, 4.36 to 28.44) *P* = .016).

## 4. Discussion

A challenge in the therapeutical management of Ménière's disease is the control of vertigo symptoms and maintaining the function of the inner ear. If conservative or less invasive treatment (grommets and Meniett device) fails to rehabilitate the equilibrium a more invasive therapy (sac surgery, canal occlusion, gentamicin, labyrinthectomy, and nerve dissection) which might lead to hearing loss has to be considered. It is commonly accepted that surgical labyrinthectomy is an effective but destructive method of preventing recurrent attacks of vertigo caused by Ménière's disease, which leads to complete deafness [5]. Because of the functional cochlear and vestibular impairment after realized labyrinthectomy, two preconditions are essential for the indication: little or no hearing impairment contralaterally and a regular contralateral vestibular function. It would be an obvious conclusion to combine the efficient but destructive labyrinthectomy with cochlear implantation to recover the auditory function of the affected ear. Former concerns that the labyrinthectomy may damage auditory neural elements with the result of no possible stimulation of the spiral ganglion by a cochlear implant could not be proved [1, 2, 7]. Nevertheless literature research concerning cases of labyrinthectomy and cochlear implantation in patients suffering Ménière's disease is limited [5] and is being performed in cases of bilateral Ménière's disease [1] or even 20 years after labyrinthectomy [6]. Zwolan et al. [2] performed to our knowledge the first simultaneous labyrinthectomy and cochlear implantation in a single patient.

As the results show, the combination of labyrinthectomy and cochlear implantation is an efficient concept for the treatment of patients with Ménière's disease and single-sided deafness in case where the above preconditions have been implemented. An excellent control of vertigo symptoms could be achieved using this therapeutical concept.

A comparison of the DHI results indicates that the functional, physical, and emotional deficits of patients with single-sided Ménière's disease develop in terms of a certain time course after simultaneous labyrinthectomy and cochlear implantation. In the first days after the operation all patients reported an increase of vertigo symptoms, such as permanent dizziness and an unsteady gait. In most cases walking was only possible with a walking aid for up to 3 days. Also the DHI score revealed a nonsignificant but indicative enhancement of physical and functional impairment. Only emotional disturbance reduced nonsignificantly immediately after surgical intervention. This could possibly be explained by the immediate emotional relief after definite intervention and the patients' belief in a successful treatment by this surgical concept. The increase of the functional and physical impairment immediately after the operation may be the result of the decompensation of a compensated system. The main preoperative complaint aside from deafness consisted of recurrent unpredictable attacks of vertigo despite two functional vestibular organs. These attacks made a satisfactory everyday life and normal social interaction impossible. The vertigo symptoms caused by the endolymphatic hydrops cannot be compensated related to unpredictable attacks. Postoperatively the per se compensated equilibrium status changed to a decompensated situation by the removal of the one-sided functional vestibular receptors. Vertigo attacks caused by endolymphatic hydrops have been treated and a restrictive but stable vestibular situation has been created, which can now be compensated by central, proprioceptive, and visual mechanisms.

This could be observed in the following course. In the first postoperative weeks, the patient's condition improved significantly. None of the patients complained about any further attacks of vertigo. Persistent feelings of dizziness developed regressively. After 6 weeks of compensation most patients reported a persistent feeling of uncertainty while walking. One patient had no more vertigo symptoms except in case of challenging physical activities. The DHI revealed a clear but still nonsignificant reduction of physical, functional, and emotional impairment compared to the postoperative status. The functional and emotional disorders also improved nonsignificantly compared to the preoperative status.

Further 6 months after surgical intervention led to a* restitutio ad integrum* in all cases. Patients reported an almost complete loss of all symptoms of vertigo and dizziness. Discomfort and a slight feeling of dizziness only occurred in situations of exhausting physical exercise or challenging physical activities. All patients have reached the stage of a well-compensated single-sided vestibular deficiency. Most patients reported a feeling of uncertainty in a dark environment. This could be explained by the lack of visual perception as one of the compensatory systems.

In the past there have been concerns that cochlear implantation after labyrinthectomy could only achieve a limited success because of an assumed impairment of auditory neural elements. But previous studies had already indicated that labyrinthectomy does not necessarily lead to a destruction of the auditory pathway [2, 7]. This study also indicated satisfying audiological results after simultaneous cochlear implantation and labyrinthectomy with a mean monosyllabic understanding at 65 dB of 69% (Table 2). These results are in contrast to the findings by others [5] comparable to regular cochlear implant patients. Since most of the patients had some degree of residual hearing preoperatively it cannot be determined whether the audiological results are related to the good condition of the intracochlear neural structures or to an advantage of the destructive procedure over others [5].

A preoperative long-term evaluation of the patients vertigo history is of importance to exclude the vestibular organs being affected bilaterally to minimize the risk of a Dandy syndrome related to a labyrinthectomized side with contralaterally impaired vestibular receptors.

## 5. Conclusion

For patients with single-sided Ménière's disease and profound sensorineural hearing loss, a simultaneous labyrinthectomy and cochlear implantation are efficient method for the treatment of vertigo and rehabilitation of the auditory system.

## Figures and Tables

**Table 1:** Time course of mean DHI score.

Quality	Preop	Postop	6 weeks	6 months
Functional deficit	11.6	18.0	11.6	1.6
Physical deficit	22.4	26.8	17.2	1.2
Emotional deficit	19.2	17.2	8.0	1.2

**Table 2:** Individual speech recognition and mean PTA (250, 500, 1000, and 2000) before and after cochlear implantation.

	Mean preop PTA	Preop monosyl at 65%	Postop numbers at 65 dB	Postop numbers at 45 dB	Postop monosyl words at 65 dB
Patient 1	87,5	0	100%	50%	55%
Patient 2	72,5	0	100%	70%	75%
Patient 3	83,75	0	100%	60%	70%
Patient 4	78,75	0	100%	90%	80%
Patient 5	87,5	0	100%	70%	65%

**Table 3:** Duration and preop frequency of attacks and presurgical treatment.

	Symptoms	Duration and preop frequency	Preop treatment
Patient 1	Rotational attacks	9 years, several times a week	Betahistine
Patient 2	Rotational attacks	26 years, 4 times a week	Betahistine, 2x sac surgery
Patient 3	Rotational attacks	3 years, 2 times a week	Betahistine
Patient 4	Rotational attacks	27 years, 20 times a day	Betahistine, lidocaine
Patient 5	Rotational attacks	8 years, several times a week	Betahistine
